# A middle aged woman presenting with massive empyema of the gallbladder: a case report

**DOI:** 10.4076/1757-1626-2-7827

**Published:** 2009-07-21

**Authors:** Olukayode Adeolu Arowolo, Oladejo Olukayode Lawal, Andrew Akinbolaji Akinkuolie, Adewale Oluseye Adisa

**Affiliations:** Department of Surgery, Obafemi Awolowo UniversityIle - Ife, Osun StateNigeria

## Abstract

Empyema of gallbladder is generally a rare disease and it is even rarer in the traditional African population where incidence of cholelithiasis is much lower compared with the Caucasian population. This is a presentation to highlight massive empyema of the gallbladder in a 58 year old woman who had no prior history of gallstone disease and who was treated with open cholecystectomy. The outcome was successful and she was followed up for a year.

## Introduction

Empyema of the gall bladder received extensive attention in the early twentieth century but has since been seldom discussed [1-3]. Until 1982, only one paper in the English medical literature dealt with the subject and it was then described as the forgotten disease [4] The paucity of literature on empyema of gallbladder in the contemporary period becomes more interesting when juxtaposed with the fact that the incidence of gallstone disease is rising particularly in the western world with reported population incidence of 20% to 50% [5,6]. While there is lack of definite information on the true population incidence and prevalence of gallstone disease in black Africa [7,8], observation similar to that in Caucasian may be true especially for the urban centres although not to the same degree of incidence. The decreasing frequency of empyema of gallbladder currently being observed worldwide may be due to early and widespread use of antibiotics and the adoption of the early cholecystectomy policy for gall bladder diseases, including acute cholecystitis [9]. It is a curiosity therefore to see a patient presenting with huge empyema of the gallbladder. This paper reported a case of massive empyema of the gallbladder and highlighted the management challenges it posed including that of emergency cholecystectomy.

## Case presentation

A 58-year-old woman, a University Lecturer of Yoruba ethnicity in Nigeria. She weighed 52 kg and 1.55 m in heght. She presented on 2^nd^ of January 2007 with 4 days history of an insidious onset of right hypochondrial colicky abdominal pain which progressed and became generalized a day prior to admission. It was associated with anorexia, nausea and one episode of non - bilious, projectile vomiting. She had dyspepsia and early satiety a couple of days prior to the onset of abdominal pain. Although she also had low grade fever there were no chills and rigor. She was never fat intolerant and had not suffered previous jaundice. She is Para 3 ^+ 0^ 3 alive woman last menstrual period was 12 years ago. She neither drink alcohol nor smoke cigerratte.

Physical examination essentially revealed an exhausted, dehydrated, tachycardic (pulse rate of 120 per minute, small volume but regular) woman in painful distress with a Blood Pressure of 140/100 mmHg. There was a firm, tender, irregularly surfaced globular abdominal mass measuring 20 cm lengthwise and extending from her right hypochondrium to the iliac fossa. Its upper limit was tucked under the rib cage and could not be reached. Although bowel sounds were hypoactive, there was no clinically demonstrable ascites.

Clinical diagnosis of infected mucocoele or empyema of the gallbladder was entertained with a differential diagnosis of infected hepatic cyst. Ultrasonography and Computerized axial tomography respectively reported mucocoele and empyema of the gallbladder with impacted stone at the neck. Her haematocrit was 39% and except for a mild azotaemia (urea level of 6.3 mmol/L), possibly due to dehydration, the electrolytes were within normal limits. Electrocardiogram showed occasional supraventricular ectopic beat.

**Figure 1.**
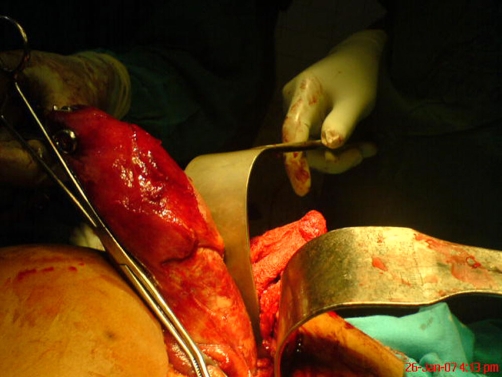
Massive gallbladder being delivered at surgery.

**Figure 2.**
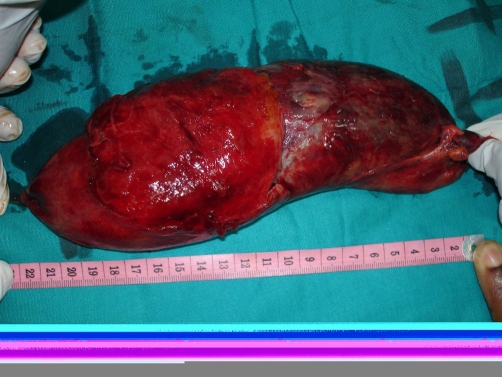
Gallbladder about 22 cm in length half the size of length of newborn baby.

She was adequately resuscitated with intravenous fluid and antibiotics and analgesic (intravenous ceftriaxone and intramuscular petazocine). She then had an emergency exploratory laparotomy and retrograde cholecystectomy. Operative findings included minimal serous ascites, massively distended and oedematous gallbladder measuring about 24 cm in longitudinal diameter (half as big as a full term baby)(Figures 1, 2). With generally glistening surface except for patches of dark discolorations of the fundus and surprisingly with no thickening of its wall. The upper third of the posterior wall of the gallbladder was completely embedded in the hepatic parenchymal. There were multiple small stones in the gallbladder and there was also an obstruction due to impacted stones at the neck and the aspirate was bilio - purulent. There were surrounding adhesions and the tissue planes were oedemtous. She had an uneventful post operative course and she had been followed up for more than a year with satisfactory outcome.

## Discussion

Gallstone pathology and the recognition that it might result in fatal complications was widely discussed during the renaissance in several treatises [10]. Some of the authors suspected then that gallstone was associated with attacks of recurrent abdominal pain. A report of the first known surgical treatment, cholecystotomy for empyema, was however not published until 1743 [10]. Empyema of the gallbladder remains a serious condition that can lead to death secondary to septicemia. Mortality as high as 25% has been reported [11]. The incidence of empyema reported in the literature varies widely, from 2.4 to 11% of cases of cholecystectomy. [10,11] Although it has been noted to be a disease of older age groups [10,11]. A higher preponderance of males has been reported in most series [11], although our patient was a middle aged woman. A preoperative diagnosis of empyema of the gallbladder can generally be difficult. The clinical presentation, physical findings, laboratory tests and even ultrasound findings (of distension, wall thickening, pericholecystic fluid collection and intraluminal sludge) may be indistinguishable from those of uncomplicated acute cholecystitis [9,10]. The reported high mortality and morbidity associated with the condition have led to emphasis being placed on early diagnosis and immediate surgical treatment [10,11].

The relatively short duration of symptoms, the low incidence of gallstone disease in our environment and the rarity of empyema of the gallbladder in general coupled with the fact the patient was a very enlightened person who could not have neglected her disease state made the clinical diagnosis slightly difficult in this case and much reliance had to be placed on the physical appearance and the location of the swelling in the abdomen.

**Figure 3.**
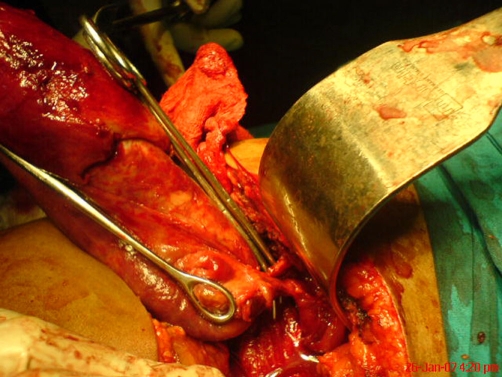
Dissection of calots triangle made easier by tissue oedema.

Performing emergency cholecystectomy on an inflamed and oedematous gallbladder with surrounding tissue oedema and adhesion is generally known to be fraught with danger of bleeding and iatrogenic injuries to structures in the triangle of Calot given the regularity and notoriety of the anatomical anomalies and variations of the extrahepatic ducts and vessels. The sheer size of the gallbladder in the case, rather than compound the problem, was found to be helpful to dissection and the operation in that the colon, duodenum and omentum were to some extent pushed down and out of the way. The splaying out occasioned by the displacement resulted in a wide triangle and the inflammatory oedema further assisted in identifying the tissue planes (Figure 3). Adoption of meticulous and cautious approach in view of the potential dangers of the operation additionally in this case.

## Conclusions

This report highlighted that gallstone diseases complicated by empyema could present without the known typical chronic clinical features of cholelithiasis that usually antedate the presentation. Absence of chronicity was in this case supported by the distensibility of the gallbladder to the enormous size encountered. The sheer size coupled with the presence of inflammatory oedematous tissue rather than compound the usual danger of operating in the condition favourably modulated the emergency open cholecystectomy offered the patient.
